# Review of Diagnostic Procedures and Approaches to Infectious Causes of Reproductive Failures of Cattle in Australia and New Zealand

**DOI:** 10.3389/fvets.2018.00222

**Published:** 2018-10-02

**Authors:** Michael P. Reichel, Lloyd C. Wahl, Fraser I. Hill

**Affiliations:** Department of Infectious Diseases and Public Health, Jockey Club College of Veterinary Medicine and Life Sciences, City University of Hong Kong, Hong Kong, Hong Kong

**Keywords:** cattle, reproductive failure, abortion, diagnosis, infectious, diagnostic approaches, Australia, New Zealand

## Abstract

Infectious causes of reproductive failure in cattle are important in Australia and New Zealand, where strict biosecurity protocols are in place to prevent the introduction and spread of new diseases. *Neospora caninum* ranks highly as an important cause of reproductive wastage along with fungal and bacterial infections. *Brucella*, a leading cause of abortion elsewhere in the world, is foreign, following successful programs to control and eradicate the disease. Leptospirosis in cattle is largely controlled by vaccination, while *Campylobacter* and *Tritrichomonas* infections occur at low rates. In both countries, Bovine Viral Diarrhea virus (BVDV) infection rates as the second most economically important disease of cattle and one that also has an effect on reproduction. Effective disease control strategies require rapid diagnoses at diagnostic laboratories. To facilitate this process, this review will discuss the infectious causes of reproductive losses present in both countries, their clinical presentation and an effective pathway to a diagnosis.

## Introduction

Infectious organisms that often cause only mild and unapparent disease, such as *Neospora caninum, Listeria monocytogenes*, Bluetongue virus (BTV), and Bovine Viral Diarrhea virus (BVDV), can cause reproductive failures when transmitted vertically from dam to offspring. Down regulation of the maternal immune system during pregnancy is necessary to prevent allogeneic rejection of the embryo. Meanwhile, the fetal immune system only starts to develop during the second trimester. Immunosuppression of the dam, coupled with the developing status of the fetal immune system, affords pathogens the opportunity to infect and grow unchecked. The mechanism of transplacental transmission of many abortifacients has not been completely defined, although there is evidence to suggest that placental macrophages may contribute to transmission of bacteria and fungi (1). Insights have also been provided by studies in mice with malaria induced abortion (2) and Campylobacter infection in sheep (3).

The response of the fetus to infection depends on the stage of gestation when infection first occurs. In the first trimester, when the fetus has no effective immune system, infectious agents kill fetal cells directly (4). If infection occurs at this stage, the calf may be born immunotolerant, as in the case of BVDV. As the fetus develops, the immune system response becomes more complete. From 98 days gestation, for example, the fetus is capable of mounting an IgG immune response against *N. caninum* (5). Within a few more days, developed bovine fetal lymphocytes are capable of mitogenic stimulation and the production of IL-2 (5). Once the immune system has matured, infection may be repelled, or, conversely, the products of inflammation may negatively affect (6) or kill the fetus (7).

In Australia and New Zealand, abortion and reproductive failure in cattle are significant limitations to cattle productivity. In the dairy industry in Australia, fetal loss accounts for between 2–3% (8) and 7% (9) of total pregnancies. Losses in New Zealand are reported to be similar, at around 6% (10). Losses in the beef industries are harder to quantify because of rangeland rearing and less supervision of animals, still, estimates of fetal losses are thought to be about half of those in the dairy industries (8). Both countries have deregulated cattle farming industries where the responsibility for diagnosing reproductive disease is the responsibility of the farmer. In cases of suspected notifiable exotic disease, government authorities will take responsibility for diagnosis and control (New Zealand^1^, Australia^2^).

The geographical isolation of these island states has allowed them to successfully implement and maintain high biosecurity protocols, maintaining freedom from Foot and Mouth disease and Bovine Spongiform Encephalopathy (BSE). It has also enabled both countries to successfully eradicate bovine brucellosis (*Brucella abortus*) (11, 12). This disease formerly had a worldwide distribution but has now been eradicated from many developed countries; although it still occurs in the USA and certain parts of Europe. Successful eradication in both countries was achieved using a combination of vaccination and compulsory test and slaughter programs. Biosecurity measures such as tail tagging and movement restrictions were also critical in the control of *B. abortus* transmission. Information on brucellosis has been extensively reviewed (13). Contagious bovine pleuropneumonia (CBPP) (*Mycoplasma mycoides*) has also been successfully eradicated in Australia (14) and New Zealand (15), while bovine tuberculosis has been eradicated from Australia (16, 17), but not from New Zealand (18). Bovine ephemeral fever virus, Q-fever (*Coxiella burnetii)* and Bluetongue virus are present within Australia (19, 20) but are absent from New Zealand^3^ (21, 22).

There are many infectious causes of bovine abortion in Australia and New Zealand. While some of these diseases are responsible for significant reproductive failures in other parts of the world, in Australia and New Zealand their clinical presentation is less severe or, occasionally, entirely benign. Table 1 summarizes these abortifacients, and their abortion record in Australia and New Zealand. Where possible, a reference has been given for the most recent evidence of disease presence, and for documented cases of abortion. The most important abortifacients in both countries, in terms of both prevalence and economic impact, are *N. caninum*, BVDV, and *Leptospira. N. caninum* infections were estimated to cost the cattle industries in both countries approximately US$ 135 million annually in 2012 (8). Seroprevalence of BVDV is high in both countries (84), and it rates as the second most economically important disease of the cattle industries (85). Multiple *Leptospira* serovars infect the countries cattle populations (86, 87). In addition to their impact on cattle reproduction, their presence poses a public health risk due to their zoonotic potential.

**Table 1:** Abortifacients in Australia and New Zealand (✓, record; ✗, no record).

**Abortifacient**	**Australia**		**New Zealand**	
	**Present**	**Abortion recorded**	**Present**	**Abortion recorded**
**PROTOZOA**				
*Neospora caninum*	✓(23, 24)	✓(25, 26)	✓(27, 28)	✓(29)
*Tritrichomonas fetus*	✓(30)	✗^a^	✓(31)	✓(32)
*Babesia bovis*	✓(33)	✓(34)	✗^b^	✗
**FUNGI**				
*Aspergillus fumigatus*	✓(35)	✓(35)	✓(36)	✓(37)
*Mortierella wolfii*	✓(38)	✓(35)	✓(39)	✓(40)
**BACTERIA**				
*Leptospira*	✓(41, 42)	✓(43, 44)	✓(45)	✓(27)
*Listeria monocytogenes*	✓(46)	✓(44)	✓(47)	✓(31)
*Listeria ivanovii*	✓(46)	✓(48)	✓(31)	✓(31)
*Campylobacter fetus* subspecies *venerealis*	✓(49, 30)	✓(50, 51)	✓(52)	✗
*Salmonella* Dublin	✓(53, 54)	✓(55, 56)	✗	✗
*Salmonella* Brandenburg	✗	✗	✓(57, 58)	✓(57)
*Bacillus licheniformis*	✓(59)	✓(60)	✓(31)	✓(31)
*Bacillus cereus*	✓(46)	✗	✓(61)	✓(62)
*Ureaplasma diversum*	✓(63, 64)	✗	✓(65)	✓(66)
*Mycoplasma bovis*	✓(67, 68)	✓(69)	✓^c^	✗
*Coxiella burnetti*	✓(20, 41)		✗(22)	✗
*Chlamydia psittaci*	✓(43)	✓(70)		✗
*Fusobacterium necrophorum*	✓(71)	✗	✓(72)	✗
*Trueperella pyogenes*	✓(73)	✓(44)	✓(74–76)	✓(77)
**VIRUSES**				
*Bovine viral diarrhea virus*	✓(78, 79)	✓(80)	✓(81)	✓(27)
*Bovine herpesvirus 1*	✓(49)		✓(82)	✗
*Bluetongue virus*	✓(19)	✗(83)	✗^*a*^	✗
*Akabane virus*	✓(19)		✗	✗
*Aino virus*	✓(43)	✗	✗	✗

a https://www.dairyaustralia.com.au/farm/animal-management/animal-health/abortion-and-infertility

b https://www.mpi.govt.nz/dmsdocument/10466-statement-absence-of-specified-diseases-from-new-zealand

c https://www.mpi.govt.nz/news-and-resources/media-releases/infected-farm-found-in-new-region/

This review focuses on the protozoa, fungi, bacteria and viruses responsible for reproductive failures of cattle in Australia and New Zealand. Discussed below are the important and interesting features of these pathogens, organized in order of importance within their phyla. The clinical presentation at necropsy is described, and the approaches required for diagnostic laboratories to make a successful definitive diagnosis of abortion etiology are outlined. Diseases that are not prevalent in these two countries are not discussed.

## Abortifacients in Australia and New Zealand

### Protozoa

#### Neospora caninum

Since its recognition as an abortifacient in cattle in the late 1980s (88), *N. caninum* has quickly become the most frequently diagnosed cause of fetal loss in cattle in Australia and New Zealand (89). Until 1990 in New Zealand, a diagnosis in a case of bovine abortion was only reached 5–16% of the time (90, 91). However, once *N. caninum* was identified as an abortifacent and histopathology of aborted fetuses undertaken more frequently, the overall diagnostic rate increased to 28% (92).

The parasite has two modes of transmission, vertical from dam to offspring, and horizontal from its definitive host, the dog, to cow. Vertical transmission is the most efficacious, while horizontal transmission plays only a minor role. The immune response in the non-pregnant cow is mediated by inflammatory cytokines that induce growth inhibition, lysis, and cell death to control the growth and spread of the organism. This inflammatory response has been proposed to play a role in inducing abortion in the pregnant cow (93). Infection late in pregnancy in a naïve dam favors transmission without clinical effect, while mid-term infection leads to abortion (94). Quantity and duration of parasitaemia, outcome of the maternal immune response and the ability of the fetus to mount an immune response against the parasite are also important factors in whether abortion ensues or not. For an excellent review on pathogenesis of Neospora abortion see Dubey et al. (95).

At necropsy, observed lesions may include thickened oedematous placental membranes, mild autolysis of the fetus, disseminated inflammatory and necrotic lesions evident by histopathology, and vascular-oriented microgliosis in the brain (96).

#### Tritrichomonas fetus

*T. fetus* is a venereal disease with a similar epidemiology to *Campylobacter fetus*, commonly transmitted from bull to cow during mating (49). *T. fetus* infection was confirmed in north-eastern Australia in the 1970's (97, 98) and New Zealand in the 1940's (32), but there are no recently published reports of disease (49). Routine screening of stud bulls used for semen collection is recommended in both countries (99), and is used to successfully prevent venereal transmission of infection by artificial insemination (100). Diagnostic testing on preputial samples is performed using bacteriological culture and molecular testing using polymerase chain reaction (PCR) (101, 102). Reproductive failures caused by *T. fetus* occur in the first half of gestation, and include early embryonic losses. In cases of abortion, lesions may include placentitis and bronchial pyogranulomatous pneumonia. The organism may be visible by histopathology of the placenta or pulmonary tissues (103), and presence can be confirmed by PCR.

#### Babesia

In Australia, *Babesia bovis* and *B. bigemina* are associated with the distribution of their tick vectors *Boophilus* and *Rhipicephalus* between 40°N and 32°S. *Babesia* species that affect cattle are not reported from New Zealand. Clinical disease in endemic regions of Australia is uncommon due to high levels of resistance, and abortion is a rare occurrence. However, pyrexia associated with infection can result in abortion of late-term pregnant cows and 6–8 weeks of infertility in bulls (34), particularly if high-risk cattle outside of endemic regions are exposed to *Babesia*. The aborted fetus may present with watery blood, jaundice, an enlarged, discolored liver and focal hemorrhage of the kidneys (34). The intraerythrocytic parasite can be visualized by Giemsa or acridine orange staining (104), and the highest concentration of parasites are found in the erythrocytes of the brain (105).

### Fungi

#### Aspergillus fumigatus

One of the most common causes of mycotic reproductive failure, abortions due to *Aspergillus fumigatus* tend to occur in the second and third trimester (35). This fungus proliferates in decomposing hay, poorly preserved silage and soil, and produces a non-airborne, pathogenic spore (106). The spores localize to the uterine caruncle and induce inflammation-induced abortion after 2–5 weeks of proliferation. On gross examination, the placenta often has swollen, necrotic cotyledons, and the intercotyledonary membrane may be diffusely thickened, wrinkled, and leather-like. Occasionally, aborted fetuses have characteristic fungal plaques, 1–10 cm in diameter involving the skin around the eyelids, neck, dorsum, and thorax (37). This is due to fungal proliferation in the amnion, penetration of the epidermis and the fetal inflammatory reaction, as well as hyperkeratosis. Mycotic abortion can be diagnosed by culture, and histologically from placental changes with visible fungal hyphae confirming infection (107).

#### Mortierella wolfii

*Mortierella wolfii* is occasionally reported in Australian cattle, and frequently reported in the North Island of New Zealand (38). The presentation of *M. wolfii* infection in pregnant cows is the same as *A. fumigatus*, except that abortion is also followed by fatal pneumonia 4–5 days later in about 20% of cases (108). Spores may be inhaled from contaminated silage, and pass into arterial circulation via the pulmonary vascular bed (109). Pathogenesis is also the same as *A. fumigatus*, and after haematogenous spread, fungal growth at the uterine caruncle causes widespread tissue necrosis and inflammation, leading to placentitis and abortion. Grossly, the placenta appears thickened, oedematous and necrotic, and other lesions are present consistent with mycotic abortion described above.

### Bacteria

A variety of bacteria species have been implicated as causes of bovine abortion, many of them causing sporadic abortion. As a general guide, if no gross lesions are observed in the aborted fetus, microbiological examination of fetal stomach content is recommended to determine if a bacterial agent is responsible.

#### Leptospira

The serovars of *Leptospira* are associated with a variety of specific reservoir hosts (110), their respective epidemiology has been extensively reviewed as causes of disease, in particular of abortions (111). A range of serovars have been identified in Australia including *L. Hardjo, Tarassovi, Pomona*, and *Szwajizak* (86, 112). In Queensland and Victoria, *L. interrogans* has been associated with reproductive disease (113, 114). In New Zealand, the first case of bovine abortion associated with leptospirosis was diagnosed in 1953 (115), and fetal losses due to leptospirosis in beef cattle are recorded at 4.7% from *L. borgpetersenii* serovar *Hardjo* and 3.6% from *L. interrogans* serovar *Pomona* (27). Mild interstitial nephritis may be seen in the aborted fetus. Although diagnosis can be made by histological observation of the organism in the heart, intestine, liver and kidney using silver staining (114), multiplex PCR or quantitative PCR (qPCR) can be performed for a rapid and comprehensive diagnostic test (116, 117, 118). Detection of *Leptospira* by isolation is not recommended, given the difficulty of culturing this organism (119).

#### Listeria

*L. monocytogenes* (120) and *L. ivanovii* (121) cross the placenta by invading bovine trophoblast cells (122) and cause abortion. Poorly preserved silage can be a source of infection and, if conditions are suitable, contamination of food and water may lead to an outbreak of abortions (120). Gross lesions in the aborted fetus may include pinpoint white foci within the liver (48). Diagnosis of *Listeria* abortion is based on isolation of *Listeria* species from culture of fetal stomach contents, lung, and liver, along with placentitis and hepatitis observed by histopathology.

#### Campylobacter fetus

Bovine genital campylobacteriosis is a venereal disease without clinical signs in infected bulls, but which causes early embryonic losses in cows. *C. fetus* subspecies *fetus* and *C. fetus* subspecies *venerealis* infections are causes of reproductive losses in Australia (49). In New Zealand, disease was last reported in 1993 (123) but is now considered absent (52). The PCR test used to detect the presence of *C. fetus* subspecies *venerealis* in New Zealand was shown to be cross-reactive with *C. hyointestinalis* (124). This is now a recognized problem (125), and its sole use is not recommended (126). A diagnosis in the adult can be made using a combination of tests (127), and multiplex PCR assays are available to check for multiple subspecies in one assay (128). Lesions in cases of abortion include pneumonia, encephalitis and inflammation of the placenta, peritoneum and liver (129). An etiological diagnosis can be made using PCR on fetal stomach contents and vaginal or uterine discharges.

#### Salmonella

*Salmonella* inhabit the intestine of their host, and infection typically occurs when cattle ingest water and feed contaminated with feces. As a result, infections often occur in outbreaks and abortion storms can sometimes ensue. Bovine abortion as a result of *S*. Dublin infection has been reported in Australia (55), while *S*. Brandenburg causes abortions in New Zealand cattle (57), as well as sheep, and has also been associated with disease in humans (130). Clinical signs of salmonellosis in cattle, besides abortion, include diarrhea, a drop in milk production, and dysentery (57, 131). *Salmonella* infects and proliferates in the placenta and, subsequently, focal necrosis is often observed in the fetal villi after abortion (132). Diagnosis can be achieved by culture of fetal stomach contents or organs, as well as serology (133).

#### Bacillus

*Bacillus* species are ubiquitous and may infect the cow from silage. Members of this genus have been reported to cause reproductive failures in Australian and New Zealand cattle (62, 134, 135), the most common of which is *Bacillus licheniformis*, followed by *B. cereus*. Abortions caused by infection with this organism usually occur in the third trimester, and sporadically amongst the herd (136). The bacterium causes a necro-suppurative placentitis and fetal broncho-pneumonia. Pneumonia and enteritis occur after the fetus swallows bacteria that infect and proliferate in the amniotic fluid. The suppuration seen microscopically can sometimes be seen grossly at post mortem. Necrotic yellow, 2–3 mm foci can be seen in the placenta, and purulent exudate may be expressed from the pulmonary airways. The leathery, dry, yellowish-brown placentitis observed with *B. licheniformis* is fairly distinctive, but needs to be differentiated from that caused by fungal infections. Diagnosis is reached by culture of the organism from stomach contents or fresh tissue (60, 137).

#### Ureaplasma diversum

*Ureaplasma diversum* is a commensal of the vagina and prepuce, and in pregnant cows an ascending infection can occur, causing abortion in the last trimester of pregnancy (138). *Ureaplasma* have also been shown to attach to spermatozoides (139) and may be spread through insemination (140). In Australia, infection in cattle has been confirmed, but without evidence of disease (63, 64), while in New Zealand, *U. diversum* has been associated with abortions and vaginitis (66). Macroscopic lesions of the placenta include thickening of large areas of the amnion and inter-cotyledonary zones of the chorio-allantois. The thickened regions are opaque white to yellow, with foci of fibrinous exudation and hemorrhage. Microscopic lesions in the placenta, and lymphocytic nodules in the fetal conjunctiva and lung are characteristic and highly suspicious of *Ureaplasma* infection (141). No gross lesions are seen in the fetus. Diagnosis can be made by multiplex PCR assay on lung and stomach content (117).

#### Mycoplasma bovis

*Mycoplasma bovis* abortion has been reported in Ireland, Germany and Australia (69, 142, 143), but despite this, clinical disease is rarely reported in Australia (67). Until 2009, there was no evidence of *M. bovis* in New Zealand dairy cattle (144), but infection was detected in 2017^4^ A potential source of transmission was recently described in Finland, when infection was introduced into previously disease-free herds by frozen semen (145). Placentitis, swollen lymph nodes, pulmonary consolidation, interstitial and bronchial pneumonia, fibrinous pericarditis and enlarged liver may be evident in the aborted fetus (142). The organism may be isolated from the stomach content (143), but diagnosis can also be made by multiplex PCR on serum, lung, and liver (117, 146, 147).

#### Coxiella burnetii

*Coxiella burnetii* is the etiological agent of Q-fever, a serious zoonotic infection that affects livestock and wild mammals worldwide, except New Zealand (148). The organism is spread in excretions of infected animals, and can be spread as aerosols or by dust. In humans, for which cattle are only rarely the source of infection (149), the disease is subclinical in >60% of the cases. In ruminants, particularly in sheep and goats, it causes abortions, as evidenced in the recent severe zoonotic outbreak in the Netherlands (150). Experimental confirmation of *C. burnetii* as the etiological agent in bovine abortion is generally lacking, complicated by the fact that *C. burnetti* is found in healthy placentas (151). To make an etiological diagnosis in cases of abortion, placentitis should be present alongside the organism, and mild to severe evidence of inflammation should be seen upon histological examination (151). Modified Ziehl-Neelsen (MZN) smears have traditionally been used to detect *C. burnetti* in the placental cotyledons, but immunohistochemistry, fluorescence *in situ* hybridization and PCR assay are also possible (151, 152).

#### Chlamydia

*Chlamydia* and *Chlamydophila* are obligate intracellular pathogens that are spread by contact with secretions and excretions of infected animals. These bacteria have been associated with abortions in cattle (153), and *Chlamydia psittaci* was isolated in a case of abortion in Australia in 1986 (70) but has not been reported since. This may have been a misidentification of a *Chlamydophila pecorum* infection (154), which does not cause abortion. In New Zealand, *C. psittaci* has been found in birds (155) but not cattle. In cases of suspected chlamydial abortions, purulent or necrotising placentitis may be observed by histopathology and genetic material may be detected by PCR (117, 156).

#### Other bacteria

A range of other opportunistic bacterial infections can lead to abortion, these include: *Escherichia coli, Fusobacterium necrophorum, Staphylococcus aureus, Streptococcus bovis, Trueperella pyogenes*, and others, which require routine diagnostic procedures to confirm the diagnosis (157).

### Viral abortion

#### Bovine viral diarrhea virus (BVDV)

The pathogenesis and diagnosis of Bovine Viral Diarrhea has recently been reviewed extensively (158). In both countries, only BVDV Type 1 infections have been recorded. In Australia, the overwhelming majority of isolates are of a Type 1c classification (159), while the diversity of strains observed in New Zealand is much greater (160). The virus has reached a high level of endemicity in both countries, and more than 80% of adult cattle usually show an antibody response. This decreases the potential for major reproductive disease but potentially devastating outbreaks of abortions can still occur when persistently infected cattle are introduced to BVDV naïve groups. The impact in both countries, and the mitigation options available have recently been reviewed (85). BVDV is immunosuppressive, increasing susceptibility to co-infection, so evidence of the virus in fetal and placental samples does not confirm BVDV as the etiological agent. Reproductive failures associated with BVDV are variable in timing and appearance, and lesions associated with BVDV are varied but may include vasculitis, placentitis, tissue necrosis, and atrophy (161–163). The virus can be detected using reverse transcription quantitative PCR (RT-qPCR) or immunohistochemistry. Diagnosis of an acute BVDV infection in the adult can be made using serology on paired serum samples.

### Endemic in either country but not causing abortion

#### Bovine herpes virus type 1 (BHV-1)

BHV-1 is a virus with a worldwide distribution, and causes respiratory and genital diseases in cattle. The syndromes are known as infectious bovine rhinotracheitis (IBR) and infectious pustular vulvovaginitis/balanoposthitis (IPV/IPB), respectively. Less commonly, BHV-1 is associated with a variety of other clinical diseases, such as encephalitis, conjunctivitis, enteritis, and a fatal systemic infection of neonatal calves. BHV-1 infection can also induce abortion, and is recognized as such in some overseas countries. Early work carried out in the mid 70's by Durham et al. (164) indicated that the viral strain causing IBR in New Zealand was unlikely to be capable of inducing bovine abortion. From recent studies, it appears that the predominant strain of BHV-1 present in New Zealand and Australia is BHV-1.2b. There is no evidence that more virulent strains of BHV-1, e.g., BHV-1.1 (associated with severe respiratory infections and abortions) or BHV type 5 (associated with neurological infections), have ever been present in New Zealand or Australia (49, 82). In cases of abortion due to infection with virulent BHV-1 subtypes found elsewhere, lesions of the aborted fetus may include inflammation and necrosis of the heart, brain, liver, kidneys, spleen, lungs, and intestine (165). For a sensitive diagnostic test, qPCR may be performed on the liver, which has the highest viral load (166).

#### Bluetongue virus (BTV)

BTV is a vector-borne virus spread by *Culicoides brevitarsis* in Australia. In New Zealand, a complete absence of *Culicoides* species coincides with the absence of BTV^5^ (167). Dependence of vector survival and distribution on environmental factors restricts BTV seasonally to the northern and southern coasts. Bluetongue disease occurs when immunologically naïve ruminants are exposed to BTV (168, 169), but disease in cattle is rare. In Australia, clinical Bluetongue disease has not been recorded in livestock to date^6^ (170). This is partly because BTV is endemic to the region, but also because the virulent BTV serotypes found in other countries are absent from Australia (170–174), and there have been no recorded BTV-induced abortions, due to their inability to cross the placenta (83). Should virulent serotypes emerge, the aborted fetus presents with hydranencephaly that increases in severity the earlier in gestation infection occurs (175). RT-qPCR performed on cerebral tissue is the most suitable diagnostic test (176, 177).

#### Akabane and aino virus

Both Akabane and Aino are members of the genus *Orthobunyavirus* and are the most pathogenic members of the Simbu serogroup. Like BTV, they are largely transmitted by the biting midge *C. brevitarsis*, and are therefore absent in New Zealand but endemic to Australia. Antibody surveys suggest a viral distribution north of the “brevitarsis line” in tropical and subtropical parts of the Australia^7^ (43, 178), associated with the known distribution of their vector (179, 180). As with other endemic viruses, clinical disease is rarely seen in this region. Early gestational exposure [between gestational day (GD) 80–105] is linked to hydranencephaly, whereas later exposure (GD 105–175) more often results in arthrogryposis (181). RT-qPCR is available for detection of, and differentiation between these viruses (182).

### Non-infectious and “undetermined” causes

Non-infectious causes of abortion include; nitrate toxicity from crops (183), isocupressic acid toxicity (184, 185), twin pregnancy, large placenta syndrome and premature shedding of fetal membranes (186), hydroallantois, drug reactions, physical trauma, inherited lethal abnormalities (187), heat stress, teratogens, iodine deficiency, and endotoxins (188). For a review see Jonker (189).

## Diagnostic approaches

Investigating abortions can be a difficult process, and there is no single diagnostic test to identify all etiologies. However, there are some common signs that indicate an infectious agent is responsible. Inflammation of the placenta, for example, occurs as a result of infection with organisms such as *U. diversum* or fungi. This results in fetal death due to a disruption of vascularity and oxygen exchange (1). Prostaglandins are also released during the inflammatory response, which results in luteolysis, and the cascade of events leading to fetal expulsion (190, 191). Once the fetus dies, the villous circulation collapses and becomes obliterated, characterized by intra-placental coagulation and endothelial disruption (192). Separation of the cotyledon from the caruncle results in cessation of the pregnancy and the fetus and membranes are expelled, manifesting as an abortion (193).

In the field, it may be necessary to obtain multiple fetuses or dam serum samples because the problem is continuing, or the cause has not been identified. Frequently there are no diagnostic clinical signs or gross lesions, so identification of the etiological agent usually requires a complete diagnostic work-up. This involves post-mortem, histopathology, microbiology, serology and, increasingly, molecular biology. Diagnostic laboratories usually focus on the most likely etiologies, and those with zoonotic potential (194). Diagnosis of the etiological agent has improved with time, from about 33–37% in the 1990s (195, 196), to 44% in the 2000s (197), to 58% (157) 2 years ago, but only if a full range of samples were collected.

**Figure 1:**
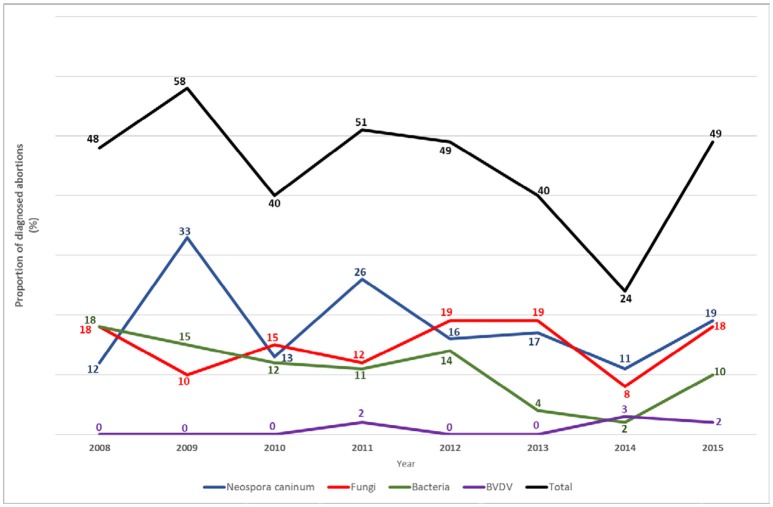
Percentage of aetiological diagnoses made per year, in 544 bovine abortion cases investigated at one veterinary diagnostic laboratory in New Zealand between 2008 and 2015.

An 8 year review (2008–2015) of 544 cases received in one commercial diagnostic laboratory in New Zealand was able to arrive at an etiological diagnosis in 45% of cases. The review showed that Neospora, followed by mycotic infections, were the most common causes of abortion (Figure 1).

The diagnostic rate is affected by:

- Submission of unsuitable samples to the diagnostic laboratory
- Delay between infection and the abortion occurring
- Destruction of the causal organism after fetal death or abortion
- Occurrence of abortion due to non-infectious causes.

Tissues and samples recommended for submission are summarized in Table 2, and discussed below.

**Table 2:** Diagnosis of infectious causes of bovine abortion in Australia and New Zealand.

**Abortifacient**	**Time of abortion**	**Samples to collect**	**Fetal and placental lesions**	**Diagnostic methods**	**OIE methods^**a**^**
**PROTOZOA**					
*Neospora caninum*	3–8 months Usually 5 months	Dam sera and fetal fluids Fixed placenta, brain, heart, liver	No gross lesions Autolysed fetus	Histopathology: granulomatous encephalitis and myocarditis Immunofluorescent antibody concentration in dam sera (>1:1,000) or any detection in fetal fluids ELISA positive dam or fetal sera	No prescribed OIE test
*Tritrichomonas fetus*	Early embryonic losses First half of gestation	Uterine discharge Fixed placenta and fetal tissues	Intercotyledonary thickening and purulence No fetal lesions	Histopathology: pneumonia, placentitis Culture PCR detection	OIE recommended tests^b^
*Babesia bovis* and *B. bigemina*	All trimesters	Dam whole blood and serum	No fetal lesions	Visualization of parasite in erythrocytes PCR detection in whole blood ELISA or IFAT on serum	OIE recommended tests^c^
**FUNGI**					
*Aspergillus fumigatus*	4 months to term	Fetal stomach contents Fixed placenta and fetal tissues	Necrotising placentitis, intercotyledonary thickening Occasional skin lesions Fetal pneumonia	Histopathology: fungal placentitis, fetal dermatitis, conjunctivitis and pneumonia Fungal culture PCR detection	No prescribed OIE test
*Mortierella wolfii*	4 months to term	Fetal stomach contents Fixed placenta and fetal tissues	Necrotising placentitis, intercotyledonary thickening	Histopathology: fungal placentitis Fungal culture PCR detection	No prescribed OIE test
**BACTERIA**					
*Leptospira*	Third trimester	Dam sera, fetal fluids and fresh tissues Fixed placenta and fetal tissues	Diffuse placentitis Fetal autolysis	Histopathology: Interstitial nephritis Visualization of typical morphology organisms in lung by silver staining, immunohistochemistry or immunofluoresence PCR detection of *Leptospira* Culture of *Leptospira* Seroconversion and serological identification by microscopic agglutination titres (four-fold increase in titres in paired sera), or high titres in non-vaccinated dams (>400) or ELISA	OIE recommended tests^d^
*Listeria monocytogenes* and *L. ivanovii*	Third trimester	Fetal fluids and fresh tissues Fixed placenta and fetal tissues	Fetal necrotizing hepatitis	Histopathology: placentitis and necrosuppurative hepatitis Culture of organism in stomach content or fresh tissue	OIE recommended tests^e^
*Campylobacter fetus* subspecies *venerealis* and *C. fetus* subspecies *fetus*	Early embryonic losses Second to third trimester	Placenta, uterine discharge, fetal fluids and fresh tissues Fixed placenta and fetal tissues	Placentitis Fetal pleuritis	Histopathology: fetal pneumonia and immunofluoresence Culture of organism (plus ELISA) and PCR detection on stomach contents and discharges	OIE recommended tests^f^
*Salmonella* Dublin and *S*. Brandenburg	Third trimester	Fetal fluids and fresh tissues Fixed placenta and fetal tissues	Placental and fetal autolysis Fetid odor	Culture of organism in stomach content or fresh tissue Histopathology: Fetal bronchopneumonia	OIE recommended tests^g^
*Bacillus cereus* and *B. licheniformis*	Third trimester	Fetal fluids and fresh tissues Fixed placenta and fetal tissues	Placentitis with hemorrhages and necrosis	Histopathology: neutrophilic placentitis, bronchopneumonia and pericarditis Culture of organism in stomach content or fresh tissue	No prescribed OIE test
*Ureaplasma diversum*	Third trimester	Fetal fluids and fresh tissues Fixed placenta and fetal tissues	Marked placentitis	Histopathology: chronic placentitis Fetal lymphocytic pneumonia and conjunctivitis PCR detection on fresh tissues	No prescribed OIE test
*Mycoplasma bovis*	Third trimester	Fetal fluids and fresh tissues Fixed placenta and fetal tissues	Placentitis, fetal pericarditis, enlarged liver Interstitial pneumonia	Immunohistochemistry of brain, liver, lung and placenta PCR detection on fresh serum, lung and liver	No prescribed OIE test
*Chlamydia psittaci*	Third trimester	Fetal fluids and fresh tissues Fixed placenta and fetal tissues	Purulent placentitis	Histopathology: placentitis PCR detection on cotyledon	No prescribed OIE test
Other bacteria	Second to third trimester	Fetal fluids and fresh tissues Fixed placenta and fetal tissues	Placentitis Fetal inflammation	Fetal inflammatory lesions Culture of organism in stomach content or fresh tissue	No prescribed OIE test
**VIRUSES**					
Bovine Viral Diarrhea Virus	Early embryonic loss All trimesters	Dam sera Fetal fluids and fresh tissues Fixed placenta and fetal tissues	Fetal cerebellar hypoplasia Porencephaly	Immunohistochemistry of fixed tissue PCR detection of virus in fetal sera or tissue Antibody in fetal fluids Dam sera: serological or virological status	OIE recommended tests^h^
Bluetongue virus	Second to third trimester	Fixed fetal tissues Fresh brain	Hydranencephaly Fetal cerebellar hypoplasia	PCR detection of virus or virus isolation in fetal sera or tissue Dam sera: Antibody detection ELISA or serum neutralization	OIE recommended tests^i^
Aino/Akabane virus	Second to third trimester	Fetal fluids and fresh tissues	Hydranencephaly and arthrogryposis Lesions absent in the cerebellum	PCR detection of virus	No prescribed OIE test

*Lesions, samples to collect and diagnostic methods for confirming pathogens*.

a http://www.oie.int/standard-setting/terrestrial-manual/access-online/

b http://www.oie.int/fileadmin/Home/eng/Health_standards/tahm/2.04.16_TRICHOMONOSIS.pdf

c http://www.oie.int/fileadmin/Home/eng/Health_standards/tahm/2.04.02_BOVINE_BABESIOSIS.pdf

d http://www.oie.int/fileadmin/Home/eng/Health_standards/tahm/2.01.12_LEPTO.pdf

e http://www.oie.int/fileadmin/Home/eng/Health_standards/tahm/2.09.06_LISTERIA_MONO.pdf

f http://www.oie.int/fileadmin/Home/eng/Health_standards/tahm/2.04.04_BGC.pdf

g http://www.oie.int/fileadmin/Home/eng/Health_standards/tahm/2.09.08_SALMONELLOSIS.pdf

h http://www.oie.int/fileadmin/Home/eng/Health_standards/tahm/2.04.07_BVD.pdf

i http://www.oie.int/fileadmin/Home/eng/Health_standards/tahm/2.01.03_BLUETONGUE.pdf

### Placenta

Collection and submission of the placenta is recommended, if it can be obtained. Careful gross examination of the placenta can be worthwhile for the observation of lesions such as necrosis and hemorrhage of cotyledons, and intercotyledonary thickening. Fresh placenta samples can be cultured for bacteria and fungi. Multiple sections of any visible lesions should be sampled and placed in 10% neutral buffered formalin. If no lesions are apparent macroscopically, collect a cotyledon and region of intercotyledonary placenta for histopathology analysis.

### Fetal samples

Fetal age can be determined from the length of the fetus in millimeters from the crown of the head to the rump (CR length) (198), or by using on-line calculators (CR length in centimeters)^8^. Fetal age should be used to classify the trimester in which the abortion occurred. This can be used to refine the likely etiologies, and guide further approaches.

#### Fixed samples

##### First trimester fetuses.

With small, first trimester fetuses (CR length up to 195 mm), the entire fetus may be placed in formalin *in toto* and dissected at the time of tissue trimming for histopathology.

##### Second and third trimester fetuses.

In second (195–600 mm CR length) and third (600–1,100 mm CR length) trimester fetuses, post-mortem examination of the fetus and collection of a standard series of tissues samples saved in 10% neutral buffered formalin for histopathology is recommended. Samples to collect include brain, lung, heart, liver, kidney, spleen, and conjunctiva.

The aborted fetus is often in a state of advanced autolysis because death occurred *in utero* some time before it was expelled. Histopathology is often still worthwhile, even on autolysed fetal tissues, and lesions in the brain can be diagnostic.

In second and third trimester fetuses, conjunctiva should be collected and assessed for any evidence of inflammation. Since the conjunctiva is a mucosal tissue in direct contact with amniotic fluid, an inflammatory response may be visible in the submucosa.

#### Fresh samples

Suitable samples to culture for the presence of bacteria or fungal abortion are aseptically collected fetal stomach contents, lung and liver. If stomach contents are available, these should be cultured first, followed by lung and then liver. Stomach content represents a sample of the ingested amniotic fluid (199, 200). Culturing placenta is only recommended if these other samples are not available (195). These fresh samples should be collected routinely, and frozen if not used immediately. Bacteria can move from the vagina to the fetus and placenta when the cervix dilates during an impending abortion. These organisms may rapidly contaminate the placenta, grow in fetal fluids, and even be swallowed by a viable fetus, thereby appearing in the stomach contents, but are unrelated to the cause of the abortion.

If skin lesions are seen on the fetus, these could be scraped and checked for the presence of fungal hyphae, used for fungal culture (195) or collected for histopathology. Fungi isolated from stomach contents are consistent with a fungal cause of abortion (201).

Serology on fetal fluids can be useful in detecting fetal antibody to *Leptospira*, BTV and BVDV (202). Fetal heart blood or fetal body cavity fluid can be used as fetal sera. The presence of specific antibodies to an infectious agent in fetal heart blood or body cavity fluid provides indisputable evidence of prenatal exposure, though this exposure may not necessarily be the cause of abortion.

Individual or multiplex PCR analyses can be performed on fresh fetal tissues. If bacteria are also isolated from the placenta or cultured from fetal stomach contents, liver or lung, this provides a highly probable positive diagnosis (203). Even more so if lesions consistent with bacterial infection are present, and other causes of abortion have been ruled out.

### Dam samples

Serum from the dam can be tested for the presence of antibodies against various infectious agents such as *Leptospira, N. caninum*, BHV-1, and BVDV (204). Rationalizing the significance of titres requires knowledge of the pre-pregnancy and pre-abortion disease status of the dam. In a previously serologically disease free herd, any subsequent positive titre would be significant. If quantitative serological values are known, these can also be used for result interpretation. For example, if *N. caninum* Indirect Fluorescent Antibody Test (IFAT) titres increase to more than 1:1,000 soon after abortion, it is indicative of etiology. The titre then generally decreases to <1:600 over a period of a few weeks. The classical serological diagnosis of leptospirosis rests on a four-fold titre rise in paired sera, which requires two sets of sera to be obtained, increasing the cost of the diagnosis. In addition, titres may be present in response to vaccination or natural infection. However, high titres with no history of vaccination would suggest an etiological diagnosis. Abortions due to BVDV infection should induce antibody titres in the dam, and might allow detection of viral antigen in the aborted fetus. Older fetuses might also have developed an antibody response to the agent themselves.

### Cost-effectiveness

The costs of the diagnostic work-up to the farmers vary a lot in Australia, with some states still subsidizing the cost of outbreak investigations. In most cases, the costs of diagnosis are fully recovered by state-run laboratories or fully charged by commercial providers. For a cost-effective laboratory investigation, submission of a full range of dam, fetal and placental samples is recommended (205). Histopathology is the best first test to employ, and, although the most expensive, it can provide important pathology clues by observation of specific lesions. This can guide subsequent serological testing to check for antibodies in dam or fetal sera against *Leptospira* spp., *N. caninum*, and BVDV. Subsequent augmentation of the diagnostic approach with microbiology and mycology enables the isolation of cultivable infectious agents. Modern serology is mostly conducted at relatively low-cost with robust and standardized commercial kits. PCR is a rapid and specific approach to diagnostics which can reduce lead times on diagnoses and can be economical in comparison to conventional techniques.

## Conclusions

In order to make a definitive diagnosis in cases of infectious abortion, the gold standard approach should include the following steps: firstly, confirm an infectious etiology is present by observing lesions and/or finding serological evidence. Secondly, identify the presence of the organism using techniques such as culture, PCR, immunohistochemistry or isolation. Finally, exclude all other possibilities. Table 2 outlines the details necessary to follow this method for the abortifacients found in Australia and New Zealand. To streamline the approach, and for cost-effectiveness, the exclusion of as many etiologies as possible should be made first by taking a detailed history, performing a post mortem examination and collecting a wide range of samples.

Useful information includes:

- The estimated abortion rate
- Whether abortion is occurring in both first-calving heifers and multiparous cows
- The estimated gestational age of aborted fetuses
- Relevant findings from physical examinations of aborting cows
- The herd's vaccination status
- Details of any weak or congenitally abnormal calves
- Whether natural or artificial breeding is used, or both
- Whether the fetus is fresh, mummified, or autolysed

This information should be used in conjunction with any characteristic post mortem findings, in order to target the tests used to identify pathogen presence. The validity of this approach holds true for new molecular diagnostic techniques that enhance our ability to detect and identify infectious organisms.

When undertaking a differential diagnosis, the unique ecology of infectious abortifacients in Australia and New Zealand should be taken into account. The notable absence of significant diseases such as brucellosis, and the absence of some pathogenic strains, serotypes and subtypes which are found in other parts of the world, should guide the approaches taken. While the possibility of emergence of exotic diseases should never be excluded, the likelihood of certain etiologies is low, and this can be taken into consideration, along with data from surveillance programs, when performing a cost-effective differential diagnosis.

A sound and systematic diagnostic process can improve the rate of diagnosis, reducing the number of abortions that cannot be reasonably attributed to a definitive cause. Molecular approaches are quickly superseding the classical culture and isolation approach, due to their increased speed, sensitivity, specificity and economy. However, presence of genetic material can rarely be used to make a diagnosis by itself, and results should be interpreted in conjunction with other techniques, and on a case-by-case basis.

## Author contributions

All authors listed have made a substantial, direct and intellectual contribution to the work, and approved it for publication.

### Conflict of interest statement

The authors declare that the research was conducted in the absence of any commercial or financial relationships that could be construed as a potential conflict of interest.

## Footnotes

^1^https://www.mpi.govt.nz/protection-and-response/responding/how-we-respond/

^2^http://www.agriculture.gov.au/biosecurity/emergency

^3^https://www.mpi.govt.nz/dmsdocument/10466-statement-absence-of-specified-diseases-from-new-zealand

^4^https://www.mpi.govt.nz/protection-and-response/responding/alerts/mycoplasma-bovis/.

^5^https://www.mpi.govt.nz/dmsdocument/10466-statement-absence-of-specified-diseases-from-new-zealand

^6^https://www.dpi.nsw.gov.au/about-us/services/laboratory-services/veterinary/bluetongue

^7^https://www.animalhealthaustralia.com.au/wp-content/uploads/AHAJ1707-NAMP-2016-17-Report_Digital_FA2.pdf

^8^http://www.ansci.wisc.edu/jjp1/ansci_repro/lab/female_anatomy/crown_rump_calculators.htm.
